# Development of an Indirect ELISA for REV gp90 Antibody Detection Using the gp90 Protein Expressed in Suspended Cells

**DOI:** 10.3390/v18010124

**Published:** 2026-01-17

**Authors:** Erjing Ke, Mengmeng Huang, Guodong Wang, Jingzhe Han, Yulong Zhang, Runhang Liu, Hangbo Yu, Ziwen Wu, Dan Ling, Xianyun Liu, Tengfei Xu, Suyan Wang, Yuntong Chen, Yongzhen Liu, Yanping Zhang, Hongyu Cui, Yulu Duan, Liuan Li, Xiaoxue Yu, Yulong Gao, Xiaole Qi

**Affiliations:** 1 Avian Immunosuppressive Diseases Division, State Key Laboratory of Animal Disease Control and Prevention, Harbin Veterinary Research Institute, The Chinese Academy of Agricultural Sciences, Harbin 150069, China; elisake0924@163.com (E.K.); huangmm1017@163.com (M.H.); wangguodong568@outlook.com (G.W.); 13132030859@163.com (J.H.); yulo_zh@163.com (Y.Z.); lrh18730280216@163.com (R.L.); yuhangbo2022@163.com (H.Y.); 17590395515@163.com (Z.W.); qingxiqingxi0831@163.com (D.L.); 13624204526@163.com (X.L.); 15083372516@163.com (T.X.); wangsuyan@caas.cn (S.W.); chenyuntong@caas.cn (Y.C.); liuyongzhen@caas.cn (Y.L.); zhangyanping03@caas.cn (Y.Z.); cuihongyu@caas.cn (H.C.); duanyulu@caas.cn (Y.D.); 2 Tianjin Key Laboratory of Agricultural Animal Breeding and Healthy Husbandry, College of Animal Science and Veterinary Medicine, Tianjin Agricultural University, Tianjin 300392, China; anliuli2003@163.com; 3 Heilongjiang Province Key Laboratory of Veterinary Immunology, Harbin Veterinary Research Institute, The Chinese Academy of Agricultural Sciences, Harbin 150069, China; 4 Jiangsu Co-Innovation Center for the Prevention and Control of Important Animal Infectious Disease and Zoonosis, Yangzhou University, Yangzhou 225009, China

**Keywords:** REV, gp90, suspension cell, ELISA, antibody detection

## Abstract

Reticuloendotheliosis virus (REV) is an immunosuppressive virus in poultry that can cause acute reticular neoplasms, chronic lymphoid tumors, stunting syndrome, and secondary infections. In many countries, the lack of effective vaccines has resulted in a high prevalence of REV infections and substantial economic losses. Enzyme-linked immunosorbent assay (ELISA)-based antibody detection is an important tool for monitoring the REV prevalence in poultry farms. ELISA coating antigens generally consist of either whole virus or viral protein; however, most commercially available REV antibody ELISA detection kits use whole virus as the coating antigen, which limits their applicability in certain diagnostic and research settings. In this study, the gp90 protein from a dominant REV strain was expressed and purified using 293F suspension cell eukaryotic expression system. Using recombinant gp90 protein as the coating antigen, an indirect ELISA for detecting gp90 antibodies (gp90-ELISA) was developed. After optimization, the optimal conditions were as follows: coating antigen concentration of 4 µg/mL with overnight incubation at 4 °C; blocking with 5% skim milk at 37 °C for 1.5 h; serum dilution of 1:200 with incubation at 37 °C for 45 min; secondary antibody dilution of 1:1000 with incubation at 37 °C for 30 min; and color development using TMB substrate at room temperature in the dark for 10 min. The cut-off value was defined as an OD_450_ ≥ 0.22 for positive samples and <0.22 for negative samples. The developed gp90-ELISA specifically detected REV-positive sera at a maximum serum dilution ratio of 1:3200. Intra- and inter-assay variation coefficients were ≤10%, indicating that the gp90-ELISA had good specificity, sensitivity, and reproducibility. Laboratory serum testing showed that the gp90-ELISA successfully detected sera from chickens immunized with the gp90 protein or infected with REV. Furthermore, analysis of clinical serum samples demonstrated 100% concordance between the gp90-ELISA results and a commercial whole-virus-coated ELISA kit. These results indicate that the gp90-ELISA is a reliable supplementary method to whole-virus-coated ELISA and has potential utility in disease surveillance and evaluation of immune responses.

## 1. Introduction

Reticuloendotheliosis virus (REV) is a member of the genus *Gammaretrovirus* within the family *Retroviridae* and shares a subfamily with mammalian C-type retroviruses. REV is one of the viruses that can cause immunosuppression in poultry [1,2,3] and is widely distributed, having spread to almost all major poultry-producing countries and regions worldwide [4]. In intensive poultry farming, REV primarily infects chickens, causing serious economic losses. Although REV can also infect ducks, geese, turkeys, peacocks, and pheasants, these species typically act as carriers without developing clinical disease [5]. REV predominantly targets the immune system, inducing various tumors in organs, such as the liver, spleen, and kidneys, leading to growth retardation, emaciation, and mortality in affected birds. REV is a single-stranded, positive-sense RNA virus. Its genome comprises three major open reading frames (ORFs)—gag, pol, and env—as well as two long terminal repeats (LTRs). The proteins encoded by these genes are essential for viral replication, assembly, and infection. The gag gene encodes core structural proteins, including matrix protein (MA), capsid protein (CA), and nucleocapsid protein (NC), which mediate virion assembly and packaging. The pol gene encodes the viral polymerase, including protease (PR), reverse transcriptase (RT), and integrase (IN), which are crucial for viral replication. The env gene encodes envelope proteins, including surface protein gp90 and transmembrane protein gp20, which mediate viral attachment to host cells. The LTRs, the repeated sequences at both ends of the genome, regulate viral integration and transcription [3,6,7].

Currently, no vaccine or effective control measures are widely available for RE in most countries. Prevention relies on early detection of REV antigens or antibodies, culling diseased birds, and flock purification. To monitor infection status, prevent viral spread, and reduce economic losses, the enzyme-linked immunosorbent assay (ELISA) is the preferred detection method due to its simplicity, speed, high sensitivity, and specificity. ELISA for serum antibodies typically uses either whole virus or viral proteins as the coating antigen. Compared to the whole virus, the viral proteins are easier to prepare at higher purity, improving assay accuracy. Moreover, new vaccine strategies such as subunit [8] and nucleic acid vaccines [9], which use a single antigen as their main component, are more likely to use ELISA with a single protein as the coating antigen for immune response evaluation and monitoring. Despite this, nearly all commercially available REV antibody ELISA detection kits currently employ whole virus as the coating antigen [10,11]. Therefore, developing a REV antibody ELISA detection kit using the main protective antigen protein as the coating antigen is of significant practical importance.

As the primary envelope protein of REV, gp90 serves as the main protective antigen. Its surface contains linear and conformational antigenic epitopes that mediate viral binding to host receptors and induce neutralizing antibody production [12,13,14]. gp90 is also a core target for genetic evolution studies, vaccine development, and diagnostic assay design [4]. In this study, the gp90 protein from a dominant REV strain was expressed and purified using a 293F suspension cell eukaryotic expression system. These gp90 proteins were used as coating antigens to develop an indirect ELISA for detecting REV gp90 antibodies (hereafter referred to as “gp90-ELISA”), providing a novel tool for REV detection and control.

## 2. Materials and Methods

### 2.1. Cells, Virus, and Plasmid, Antibody, and Serum

293F and 293T cells were maintained by the Avian Immunosuppressive Disease Division at the Harbin Veterinary Research Institute, Chinese Academy of Agricultural Sciences (hereinafter referred to as “our lab”). The representative REV strain HLJR0901 (GenBank accession number GQ415646) [15,16] was isolated and characterized in our lab. The recombinant eukaryotic expression plasmid p19-2-6his-peptide-gp90-12his, which expresses the gp90 protein of the HLJR0901 strain, was also constructed in our lab. The following antibodies and sera are also from our lab: a monoclonal antibody (MAb) against REV gp90 [17]; REV-positive and REV-negative sera; and positive sera of Marek’s disease virus (MDV), chicken infectious anemia virus (CAV), avian metapneumovirus (aMPV), avian adenovirus (FAdV), avian leukemia virus (ALV), avian reovirus (ARV), and infectious bursal disease virus (IBDV). The positive sera for avian influenza virus (AIV), Newcastle disease virus (NDV), and infectious bronchitis virus (IBV) were obtained from Harbin Weike Biotechnology Co., Ltd. (Harbin, China). Horseradish peroxidase (HRP)-conjugated rabbit anti-chicken immunoglobulin G (IgG) and fluorescein isothiocyanate (FITC)- conjugated rabbit anti-chicken IgG secondary antibodies were purchased from Sigma (Livonia, MI, USA).

### 2.2. Main Reagents and Instruments

Polyjet in vitro DNA transfection reagent (Signagen, Frederick, MD, USA); Polyethyleneimine (PEI) (Beyotime, Shanghai, China); DMEM liquid culture medium, 293pro CD 293M serum-free culture medium (Shanghai Yuanpei Biotechnology Co., Ltd., Shanghai, China); fetal bovine serum, skim milk powder (Sigma, Livonia, MI, USA); Ni Sepharose 6 Fast Flow (GE, Boston, MA, USA); TMB substrate (TIANGEN, Beijing, China); PageRuler protein marker, Pierce BCA protein quantitation kit (Thermo Scientific, Waltham, MA, USA); phosphate-buffered saline (PBS) buffer (powder) (Coolaber, Beijing, China); Tween-20 (Solarbio, Beijing, China). The commercial kit used as a detection control was an avian REV ELISA antibody test kit coated with whole virus (hereinafter referred to as REV-ELISA). ELISA plates (Costar, Corning, NY, USA) and an automatic enzyme-linked immunosorbent assay instrument (Model Elx800, BioTek, Winooski, VT, USA) were used.

### 2.3. Expression and Purification of REV gp90 Protein in Suspension Cells 293F

The recombinant eukaryotic plasmid p19-2-6his-peptide-gp90-12his was transfected into suspension-cultured 293F cells for expression of the REV gp90 protein. When the cell density reached 2 × 10^6^/mL, transfection was performed using 20 µg of plasmid per 30 mL of culture, with a plasmid-to-PEI ratio of 1:1.5. Briefly, 20 µg of plasmid DNA and 30 µL of PEI were separately diluted in 1 mL of culture medium and preincubated. The PEI solution was then added to the plasmid solution, and mixed thoroughly, and incubated at room temperature for 15 min to allow complex formation. Finally, the mixture was added dropwise to the cell culture flask, and the cells were incubated at 37 °C with agitation at 112 rpm in a humidified atmosphere containing 5% CO_2_. Cell culture supernatants were harvested 4 days post-transfection.

The harvested supernatant was purified using Ni Sepharose affinity chromatography. One milliliter of Ni Sepharose resin was added to the cell supernatant and incubated overnight at 4 °C with gentle rotation to allow protein binding. The resin was loaded onto a gravity-flow column, and the flow-through was collected. The column was sequentially washed with phosphate buffer containing 20, 40, and 60 mM imidazole to remove nonspecifically bound proteins. Finally, the purified REV gp90 protein was eluted with phosphate buffer containing 250 mM imidazole. Protein purity and identity were analyzed by sodium dodecyl sulfate-polyacrylamide gel electrophoresis (SDS-PAGE) and Western blot. The primary antibody used was the REV gp90 MAb (1:1000 dilution), and the secondary antibody was the IRDye800CW-labeled goat anti-mouse IgG (1:10,000 dilution). Protein concentration was determined using the Pierce BCA Protein Assay Kit.

### 2.4. Establishment and Optimization of gp90-ELISA

The checkerboard titration method was used to determine the optimal antigen coating concentration and serum dilution for the ELISA. REV-gp90 was serially diluted in carbonate buffer to final concentrations of 32, 16, 8, 4, 2, and 1 µg/mL. Aliquots of 100 µL per well were added to 96-well ELISA plates and incubated overnight at 4 °C for coating. Plates were washed three times with PBS containing 0.05% Tween-20 (hereinafter referred to as PBST), and then blocked with PBS containing either 5% skim milk or 5% bovine serum albumin (200 µL per well) at 37 °C for 0.5, 1, 1.5, 2, 2.5, and 3 h. After washing once with PBST, REV-positive and negative sera were diluted to 1:100, 1:200, 1:400, 1:800, 1:1600, 1:3200, and 1:6400. Each dilution (100 µL per well) was incubated at 37 °C for 30, 45, or 60 min. Plates were then washed five times with PBST, followed by incubation with HRP- conjugated rabbit anti-chicken IgG diluted to 1:1000, 1:2000, 1:3000, 1:4000, 1:5000, 1:6000, 1:7000, and 1:8000 (100 µL per well) at 37 °C for 30, 45, or 60 min. After five additional washes with PBST, 100 µL of TMB substrate was added to each well and incubated in the dark at room temperature or 37 °C for 10, 15, or 20 min. The enzymatic reaction was terminated with 2 M sulfuric acid, and absorbance was measured at 450 nm (OD_450_). Optimal assay conditions were defined as those yielding the maximum ratio of the optical density of positive serum samples to that of negative samples (P/N). After identifying the optimal antigen coating concentration and serum dilution, the optimal antibody dilution, blocking conditions, serum incubation time, and antibody incubation time, as well as TMB incubation time and temperature, were subsequently determined.

### 2.5. Determination of the Cut-Off Value

A total of 294 REV-negative chicken serum samples were collected. The OD_450nm_ values of these serum samples were measured using the preliminarily established gp90-ELISA and used to calculate the cut-off value. The cut-off value was determined using the following formula: Cut-off value = mean OD_450nm_ of negative samples ($\bar{x}$) + 3 × standard deviation (SD).

### 2.6. Specificity Test

The specificity of the gp90-ELISA developed in this study was evaluated by comparing the cut-off value with the OD_450nm_ values of the positive sera for common avian pathogens, including MDV, CAV, ALV, AMPV, ARV, IBDV, FADV, AIV, NDV, and IBV. Serum samples were diluted 1:200, and each sample was tested in triplicate. The mean OD_450nm_ values of each sample were calculated.

### 2.7. Sensitivity Test

To determine the sensitivity of this ELISA detection method, the REV antibody positive serum was serially diluted to 1:100, 1:200, 1:400, 1:800, 1:1600, 1:3200, 1:6400, 1:12,800, and 1:25,600. These dilutions were tested using the gp90-ELISA developed in this study. In parallel, the samples were analyzed using a commercial REV-ELISA kit and an indirect immunofluorescence assay (IFA) for comparison. The commercial REV-ELISA was performed according to the manufacturer’s instructions, using an antibody titer of 1071 as the cut-off value; samples with titers ≥ 1071 were considered positive for REV antibodies. For the IFA, 293T cells were transfected with the plasmid p19-2-6his-peptide-gp90-12his using PolyJet transfection reagent. The culture medium was replaced after 5 h, and the cells were fixed with pre-cooled 4% paraformaldehyde for 30 min after 24 h post-transfection. After washing with PBS, the cells were blocked with 5% skim milk at 37 °C for 1 h. REV-positive serum diluted from 1:100 to 1:6400 in PBS was used as the primary antibody, and incubation at 37 °C for 1 h, followed by washing with PBS. FITC-conjugated rabbit anti-chicken IgG was then added and incubated for 45 min, followed by five washes with PBS. Specific-pathogen-free (SPF) chicken serum served as the negative control. Fluorescence signals were observed using an inverted fluorescence microscope.

### 2.8. Reproducibility Test

To evaluate the reproducibility of the developed gp90-ELISA, two independent batches of purified REV-gp90 protein were used as coating antigens for intra-batch and inter-batch assessments, respectively. Three REV-positive serum samples were selected for detection using gp90-ELISA, with each serum sample tested in triplicate. The OD_450nm_ values were recorded, and the mean and SD were calculated. The coefficient of variation (CV) was calculated using the following formula: CV = (SD/$\bar{x}$) × 100%.

### 2.9. Laboratory Serum Sample Detection

To evaluate the practicality of the gp90-ELISA detection method, this study prepared laboratory samples using two animal experiments. In one animal experiment, the emulsified REV gp90 protein was administered intramuscularly to seven 5-week-old chickens at a dose of 5 μg per bird. A booster inoculation using the same method and dosage was performed 14 days after the primary immunization. Serum samples were collected 14 days after the first and second immunizations. In another animal experiment, seven 3-week-old chickens were inoculated intraperitoneally with REV (HLJR0901 strain) at a dose of 1 × 10^4^ TCID_50_ per chicken. A booster inoculation was administered 14 days after the primary inoculation using the same method and dosage. Serum samples were collected 14 days after the first and second inoculations. All serum samples were tested for antibodies using the gp90-ELISA established in this study and a commercial REV-ELISA. For samples showing inconsistent results between the two assays, indirect IFA was performed for confirmation.

### 2.10. Clinical Serum Sample Detection

A total of 134 clinical chicken serum samples were tested using the gp90-ELISA developed in this study and a commercial REV-ELISA. These sera were collected by farm technicians and sent to our lab for analysis. They originated from six chicken farms across three provinces with suspected immunosuppression symptoms and included broiler chickens and laying hens aged from 1 day to 12 weeks (Table 1).

### 2.11. Statistical Analysis

All data generated in this study were obtained from at least three independent experiments and are presented as mean values. Statistical analyses were performed using GraphPad Prism software (version 9.0; GraphPad Software, San Diego, CA, USA).

## 3. Results

### 3.1. Preparation and Identification of REV gp90

**Figure 1:**
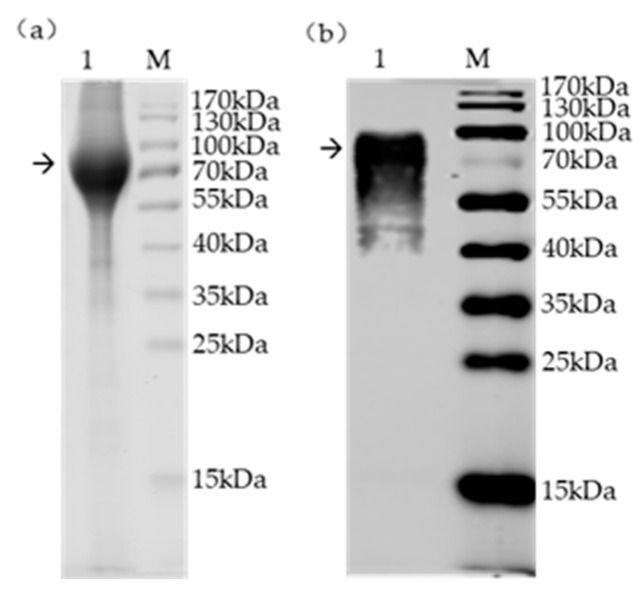
Expression and Purification of REV gp90 protein. (**a**) SDS-PAGE analysis; (**b**) Western blot identification. M: Protein molecular weight marker; 1: Purified gp90. The gp90 protein washighlighted with an arrow.

SDS-PAGE analysis showed that the gp90 protein was successfully expressed and purified after nickel column purification (Figure 1a). Western blot analysis confirmed that the expressed gp90 protein could be recognized by REV gp90 MAb (Figure 1b). The gp90 protein exhibited a relatively high molecular weight (approximately 80 kDa), likely attributable to post-translational modifications in eukaryotic cells. The final concentration of the purified REV gp90 protein reached 731.91 µg/mL.

### 3.2. Establishment and Optimization of the gp90-ELISA

In this gp90-ELISA, the recombinant eukaryotically expressed gp90 protein was used as the coating antigen for ELISA development. Optimal assay conditions were determined by checkerboard titration as follows: coating concentration of 4 µg/mL with overnight incubation at 4 °C; blocking with 5% skim milk at 37 °C for 1.5 h; serum dilution of 1:200 with incubation at 37 °C for 45 min; secondary antibody dilution of 1:1000 with incubation at 37 °C for 30 min; and color development with TMB in the dark at room temperature for 10 min. All experiments were performed in triplicate. Using 294 negative serum samples, the cut-off value was calculated based on the mean and SD of the OD values. The mean OD was 0.105, with an SD of 0.037. Accordingly, the cutoff value was set at 0.22. Samples with OD_450_ ≥ 0.22 were considered positive for REV gp90 antibodies, whereas samples with OD_450_ < 0.22 were considered negative.

### 3.3. Results of Specificity Test

**Figure 2:**
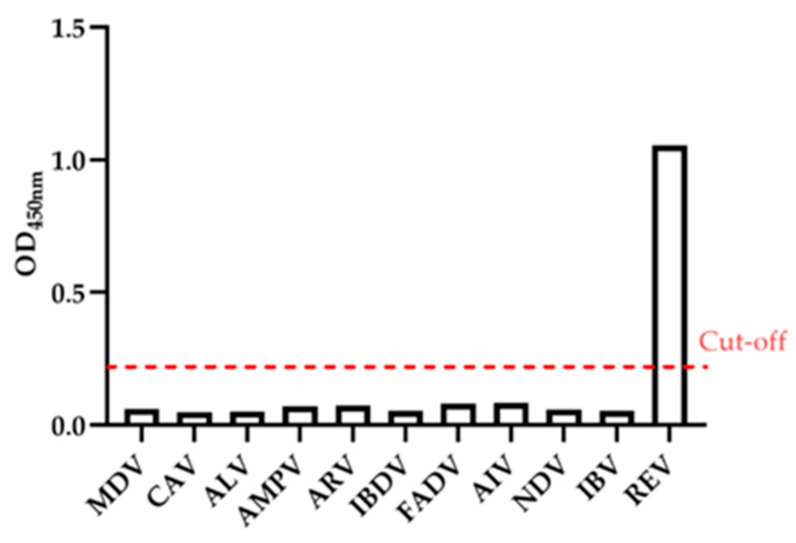
Specificity Detection of gp90-ELISA. The cut-off value for gp90-ELISA (OD_450nm_, 0.22) is marked.

Sera positive for common avian pathogens MDV, CAV, ALV, AMPV, ARV, IBDV, FADV, AIV, NDV, and IBV, all yielded OD_450nm_ values below 0.22, and were therefore classified as negative by the gp90-ELISA. In contrast, REV-positive serum produced an OD_450_ value greater than 0.22 and was correctly identified as positive. These results indicate that the gp90-ELISA exhibits high specificity (Figure 2).

### 3.4. Results of Sensitivity Test

**Figure 3:**
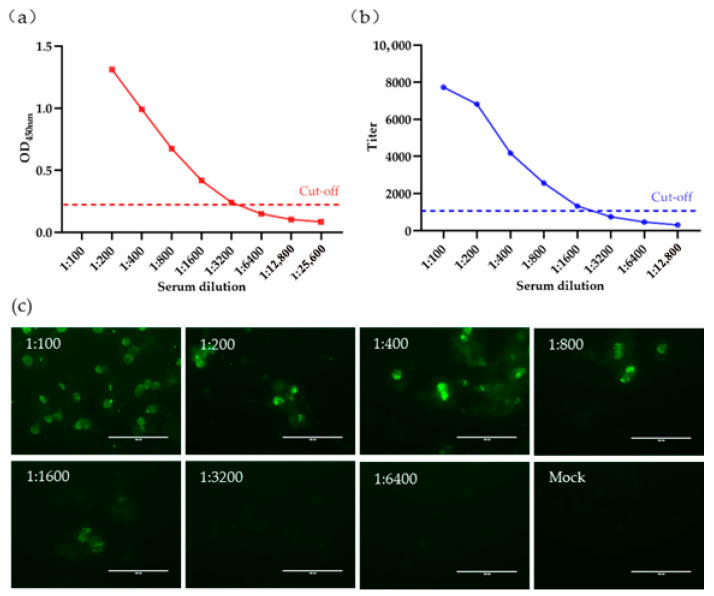
Sensitivity Detection of gp90-ELISA. (**a**,**b**) Comparison of the sensitivity of gp90-ELISA (**a**) and the commercial REV-ELISA (**b**) in detecting the same REV-positive serum. The cutoff values for gp90-ELISA (OD_450nm_, 0.22) and REV-ELISA (titer, 1071) are displayed; (**c**) Sensitivity detection of the same REV-positive serum in (**a**,**b**) using indirect immunofluorescence assay (IFA). The scalebar in each figure represents 100 µm.

The sensitivity of gp90-ELISA was evaluated and compared with that of the commercial REV-ELISA. The maximum serum dilution detectable by gp90-ELISA was 1:3200 (Figure 3a), whereas the commercial ELISA, which uses whole virus as the coating antigen, detected serum up to a dilution of 1:1600 (Figure 3b). Similarly, the maximum dilution of the same serum detectable by IFA was 1:1600 (Figure 3c). These findings indicate that the gp90-ELISA demonstrates superior sensitivity.

### 3.5. Results of Reproducibility Test

The intra-batch or inter-batch CVs for REV antibody-positive serum samples tested using two independent batches of gp90-coated plates were both below 10%, indicating good assay reproducibility (Table 2).

### 3.6. Results of Laboratory Serum Sample Detection

**Figure 4:**
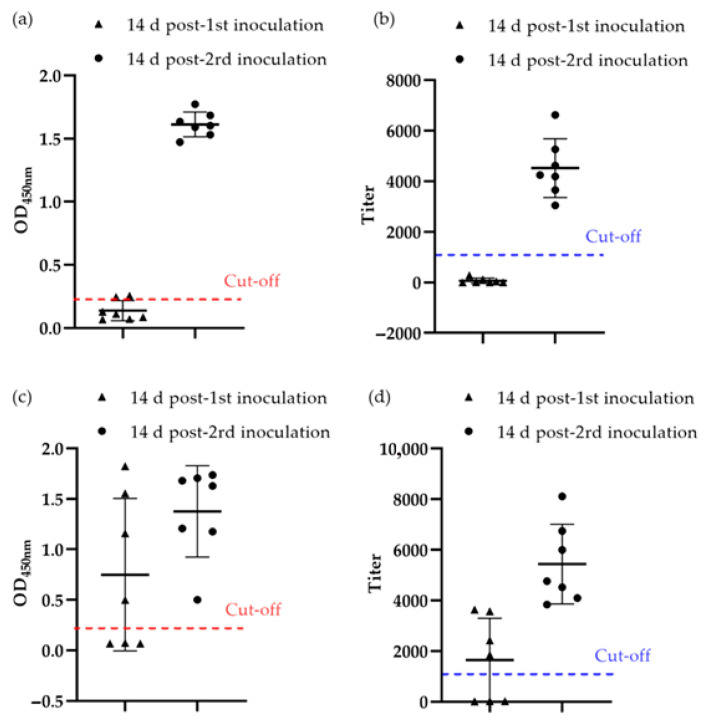
Detection of serum antibodies in laboratory samples. (**a**,**b**) Detection of serum antibodies of chickens inoculated with gp90 protein by gp90-ELISA (**a**) and the commercial REV-ELISA (**b**); (**c**,**d**) Detection of serum antibodies of chickens inoculated with REV by gp90-ELISA (**c**) and the commercial REV-ELISA (**d**). The cut-off values for gp90-ELISA (OD_450nm_, 0.22) and REV-ELISA (titer, 1071) are marked.

In chickens inoculated with the gp90 protein, the gp90-ELISA established in this study detected a seropositive rate of 29% (2/7) at 14 days post-1st inoculation and 100% (7/7) at 14 days post-2nd inoculation (Figure 4a). Conversely, the commercial REV-ELISA detected 0% (0/7) seropositivity at 14 days post-1st inoculation and 100% (7/7) at 14 days post-2nd inoculation (Figure 4b). Two serum samples that were weakly positive by gp90-ELISA but negative by commercial REV-ELISA were confirmed to be REV antibody–positive by IFA.

In chickens inoculated with REV, the gp90-ELISA detected seropositive rates of 57% (4/7) at 14 days post-1st inoculation and 100% (7/7) at 14 days post-2nd inoculation (Figure 4c), which were consistent with the results obtained using the commercial REV-ELISA (Figure 4d).

### 3.7. Results of Clinical Serum Sample Detection

**Figure 5:**
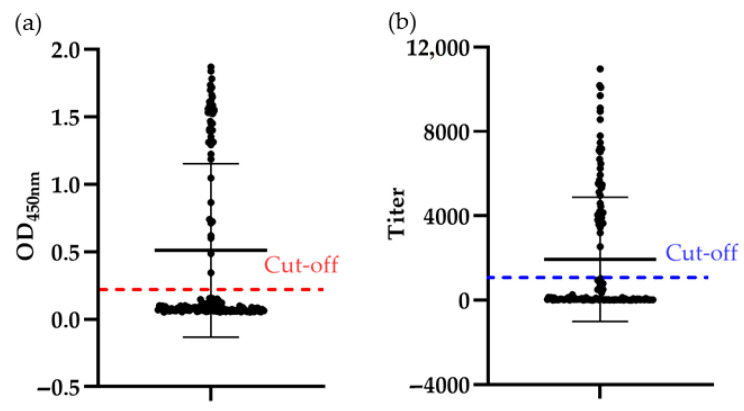
Antibody detection of clinical serum samples. (**a**) gp90-ELISA; (**b**) the commercial REV-ELISA. The cut-off values for gp90-ELISA (OD_450nm_, 0.22) and REV-ELISA (titer, 1071) are marked.

A total of 134 clinical chicken serum samples were tested using the gp90-ELISA established in this study and the commercial REV-ELISA. Forty-five samples were positive, and 89 samples were negative by both methods (Figure 5). The overall concordance rate between the two assays was 100% (134/134) (Table 3).

## 4. Discussion

In many countries, REV infections are highly prevalent and cause substantial economic and health impacts due to the lack of effective vaccines. Antibody detection is an important approach for monitoring the prevalence of REV in poultry farms, and ELISA offers a significant advantage for high-throughput antibody screening. ELISA for detecting antibodies can be classified according to the coating antigen used, including whole-virus antigens and viral protein antigens. Compared with whole-virus antigens, viral proteins offer several advantages. First, viral proteins can be purified more readily to a higher purity, which is critical for improving assay accuracy and specificity. Second, viral proteins can be produced using recombinant DNA technology, enabling large-scale production with consistent quality while reducing the risk of contamination by other viral components [10]. Despite these advantages, most commercially available ELISA kits for detecting antibodies against REV currently use the whole virus as the coating antigen. This limitation motivated the present study to evaluate the feasibility of using viral proteins, particularly the gp90 protein, as an ELISA coating antigen. The gp90 protein was chosen because it is an important component of the REV envelope and exhibits strong antigenic specificity and immunogenicity, making it an ideal candidate for detecting REV infection [8,18]. Moreover, as gp90 is a key antigen in the development of novel vaccines, such as sub-unit and nucleic acid vaccines, an ELISA based on gp90 would be valuable for evaluating and monitoring vaccine-induced immune responses.

The purity and stability of the coating antigen are critical determinants of protein-coated ELISA performance [19]; therefore, protein preparation and purification are essential steps. Several studies have expressed REV gp90 protein using prokaryotic expression systems [8,20], which are rapid, efficient, and cost-effective. However, prokaryotic hosts such as *Escherichia coli* lack the post-translational modification mechanisms of eukaryotic cells and are unable to glycosylate or phosphorylate the expressed proteins. Moreover, after purification, recombinant proteins expressed by prokaryotic expression systems may retain residual host proteins, potentially leading to non-specific reactions and false-positive results.

For viral membrane proteins, eukaryotic expression systems more closely mimic the native cellular environment and can produce biologically active proteins [21,22]. Compared with prokaryotically expressed gp90, the gp90 protein expressed by eukaryotic expression systems has advantages in detecting REV infections and developing vaccines [18]. However, glycosylation patterns in insect or yeast cells differ from those in mammalian cells, which may affect protein function. Moreover, yeast cultivation carries a risk of spore dissemination requiring additional biosafety precautions. Mammalian cell lines more closely approximate the natural expression environment of viral proteins and may yield proteins with higher activity and stability (Table S1). 293F cells are commonly used with efficient gene delivery systems and are characterized by rapid growth, high transfection efficiency, and stable expression of exogenous proteins [23], making them suitable for large-scale protein production in the biopharmaceutical industry. In this study, a suspension culture of 293F cells was used to express REV gp90 protein. After optimization of expression conditions, the REV gp90 protein was secreted into the supernatant, facilitating downstream collection and purification. High-purity protein was obtained using standard affinity chromatography techniques, providing a reliable material basis for its application as a coating antigen in ELISA. Nevertheless, an avian cell expression platform may more closely reflect the native host environment of avian viruses and could further improve protein quality. The development of stable avian cell lines expressing viral proteins may therefore represent a promising direction for future preparation of REV gp90 protein.

The gp90-ELISA developed in this study exhibits good specificity and sensitivity. This assay specifically detects antibodies against REV gp90 and shows no cross-reactivity with antibodies to other common viruses, ensuring accuracy of the detection results. In sensitivity testing, the gp90-ELISA achieved a detectable dilution of 1:3200, outperforming the commercial REV-ELISA kit (1:1600). Notably, the result formats differ between the two assays, with the gp90-ELISA reported as OD_450nm_ values and the commercial REV-ELISA kit expressed as titers. Additionally, optimization of experimental conditions identified the optimal coating antigen con-centration and serum dilution. Preliminary reproducibility was confirmed through intra-batch and inter-batch testing using two batches of gp90-coated plates; however, additional batches should be evaluated to further validate consistency. These parameters represent essential components of reagent stability assessment. Nevertheless, the existing data only support initial laboratory development and are insufficient for a diagnostic product intended for market approval. The assay remains at an early development stage. Future work will focus on comprehensive stability evaluations in accordance with product maturation standards and regulatory requirements, including storage stability, freeze–thaw stability, or coated-plate stability.

To further evaluate the practical applicability of the gp90-ELISA, laboratory serum samples were tested. In chickens immunized with the gp90 protein, gp90-ELISA results showed that the positivity rate of REV gp90 antibodies increased progressively with successive immunizations and ultimately reached 100%, indicating that the assay may be suitable for evaluating subunit vaccine immune responses. In REV-inoculated chickens, gp90-ELISA results were completely consistent with those of the commercial REV-ELISA detection results, indicating that gp90-ELISA may also be applied to evaluate whole-virus vaccine–induced immunity or for preliminary screening of REV infection in poultry flocks. In addition, testing of laboratory serum samples suggested that the gp90-ELISA may exhibit an advantage in detecting weakly positive antibodies; however, further clinical testing is required for optimization and validation. Analysis of clinical serum samples demonstrated 100% agreement between the gp90-ELISA and commercial REV-ELISA. Laboratory and clinical results further indicated that the gp90-ELISA can specifically detect serum antibodies across a range of titers, supporting its good specificity and sensitivity. Although other gp90-based ELISA methods coated with yeast–expressed gp90 protein [18] or p30-gp90 fusion proteins [20] have been reported, they have not been commercialized; direct comparison with the assay developed in this study is not feasible. Nevertheless, expanded testing with additional clinical samples and further comparative evaluations are necessary to advance the maturation and validation of this gp90-ELISA. ELISA using recombinant protein as coating antigen and those using whole virus as the coating antigen each possess distinct characteristics. From the perspective of antigen preparation, recombinant proteins are generally more efficient and safer than whole viruses, while both approaches complement each other in antibody detection and application scenarios. With continued optimization, the gp90-ELISA developed in this study is expected to become a valuable supplementary tool for REV detection and diagnosis.

REV has only one serotype [24]. Antigenic subtypes identified based on MAb reactivity exhibit distinct but minor differences in neutralization titers [25]. The HLJR0901 strain used in this study is a representative REV isolate from China and shares 99–100% amino acid sequence homology in the gp90 gene with prevalent strains in North America and other regions [16]. Therefore, the gp90-ELISA developed in this study is suitable for the universal detection of REV gp90 antibodies, but cannot differentiate antibodies against distinct REV subtypes. In future studies, subtype-specific sera or MAbs will be generated to further evaluate and validate the universal detection capability of this gp90-ELISA. If MAbs with subtype-discriminatory significance can be identified, competitive or blocking ELISAs based on these MAbs could also be developed for the identification and detection of REV subtype antibodies.

## 5. Conclusions

This study successfully developed a gp90-ELISA for detecting REV gp90 antibodies using viral proteins prepared in a eukaryotic cell suspension culture system as coating antigens. The assay is characterized by high specificity and sensitivity, effectively detects antibodies in gp90- or REV-inoculated chickens, and demonstrates promising applications in clinical sample detection. This gp90 ELISA can serve as a beneficial supplement to the whole REV-coated ELISA and could play an important role in disease surveillance and immune efficacy evaluation.

## Figures and Tables

**Table 1:** Clinical serum samples used in this study.

Batch	Samples Number	Breed	Age	Source	Sampling Year
1	20	Layer	12-week-old	Beijing	2025
2	34	Broiler	1-day-old	Heilongjiang	2025
3	34	Broiler	7-week-old	Heilongjiang	2025
4	10	Layer	10-week-old	Hebei	2025
5	14	Layer	8-week-old	Heilongjiang	2025
6	22	Layer	6-week-old	Heilongjiang	2025

**Table 2:** The intra batch and inter batch coefficients of variation in gp90-ELISA.

Sera	Intra Batch 1		Intra Batch 2		Inter Batch	
	$\bar{x}$ ± SD	CV%	$\bar{x}$ ± SD	CV%	$\bar{x}$ ± SD	CV%
REV-1	1.445 ± 0.006	0.4%	1.652 ± 0.022	1.3%	1.549 ± 0.114	7.4%
REV-2	1.616 ± 0.003	0.2%	1.728 ± 0.028	1.6%	1.672 ± 0.064	3.8%
REV-3	1.721 ± 0.014	0.8%	1.852 ± 0.038	2.1%	1.786 ± 0.076	4.3%

**Table 3:** Detection of clinical samples using the gp90-ELISA and the commercial REV-ELISA kit.

gp90-ELISA	REV-ELISA		
	Positive	Negative	Total
Positive	45	0	45
Negative	0	89	89
Total	45	89	134
Coincidence	100%	100%	100%

## Data Availability

Data can be requested by writing to the author.

## Supplementary Materials

The following supporting information can be downloaded at: https://www.mdpi.com/article/10.3390/v18010124/s1, Table S1: Comparison of different protein expression sys-tems.
