# Supplementary material for: Development of an Indirect ELISA for REV gp90 Antibody Detection Using the gp90 Protein Expressed in Suspended Cells
Source: Viruses. 2026 Jan 17;18(1):124. doi: 10.3390/v18010124 (PMC12846372; doi:10.3390/v18010124)
Supplement: Supplementary file 1 [file viruses-18-00124-s001.zip › viruses-4062103-supplementary.pdf]

Table S1. Comparison of different protein expression systems

Expression system	Post-translational modifications	Biosafety risks
Prokaryotic system（*E. coli.*）	Lack	Low
Pichia pastoris	Risk of hyper-glycosylation	Risk of spore expansion
Baculovirus / Insect cells	Risk of incomplete glcosylation	Low
Mammalian cells (293F)	Closer to viral living environment	No
